# Clinical Scenarios of the Application of Electrical Impedance Tomography in Paediatric Intensive Care

**DOI:** 10.1038/s41598-019-41774-1

**Published:** 2019-03-29

**Authors:** Patrick Davies, Samra Yasin, Simon Gates, David Bird, Catarina Silvestre

**Affiliations:** Paediatric Critical Care Unit, Nottingham Children’s Hospital, Nottingham University Hospitals NHS Trust, Nottingham, NG7 2UH UK

## Abstract

EIT is a radiation-free functional modality that enables bedside imaging and monitoring of lung function and expansion. Clinical interest in this method has been driven by the need for bedside monitoring of the dynamics of the lungs and the effects of ventilatory manoeuvres, including changes in ventilator settings, suctioning, chest drains, positioning and physiotherapy. We aimed to describe the use of Electrical Impedance Tomography (EIT) as a clinical tool in a tertiary Paediatric Intensive Care unit. Children requiring intensive care with a variety of clinical conditions had an electrode belt with 16 electrodes wrapped around the chest, which sequentially applied a small alternating current from each electrode pair. The signal gives information on both real time, regional, global, and relative data. With the correct application, and understanding of the monitor, much clinical information can be gained, with potentially significant patient benefit. We present the clinical use of EIT in six conditions: Asthma, Ventilation weaning and expansion recoil, Sequential Lobar Collapse, Targeted Physiotherapy, Pleural Effusion assessment, and PEEP optimisation. Screenshots and analyses are offered displaying the pragmatic use of this technology. Electrical Impedance Tomography is a clinically useful tool on the Paediatric Intensive Care unit. It allows monitoring of a patient’s respiratory function in ways which are not possible through any other means. An understanding of respiratory physiology will allow use of this information to improve patient outcomes.

## Introduction

Electrical Impedance Tomography (EIT) is a radiation-free functional modality that enables bedside imaging and monitoring of lung function and expansion. It has been evaluated in a number of pulmonary conditions in humans and animal models^1–8^. It has been used in various clinical settings including acute respiratory distress syndrome (ARDS), establishing the best positive end expiratory pressure (PEEP)^8–12^, the response of the lungs to recruitment manoeuvres^12–16^ and trying to minimize areas of collapse and hyperinflation^6,17^. There are some studies that evaluate the application of high-flow nasal cannula therapy^18,19^ and quantify the extent of pulmonary oedema in acute lung injury^20^.

EIT however does not offer the same spatial resolution when compared with modalities such as Computerised Tomography (CT) scanning. It does however offer good temporal resolution and informs clinicians on real time regional ventilation distribution. The images produced can also be affected by body movement, changes in electrode contact, changes in posture^21^, as well as interference with other medical devices^22^. These need to be considered when interpreting the images.

Clinical interest in this method has been driven by the need for bedside monitoring of the dynamics of the lungs and the effects of ventilatory manoeuvres, including changes in ventilator settings, suctioning, chest drains, positioning and physiotherapy.

Our aim is to disseminate our experience of the use of Electrical Impedance Tomography in the use of patients in Paediatric Intensive Care, to act as a clinical primer for other clinicians and to communicate the pragmatic use of this technology.

## Methods

An electrode belt with 16 electrodes is wrapped around the chest, which sequentially apply a small AC current from each electrode pair. The impedance between the electrodes sending the current and the receiving electrodes will change dependant on the makeup of the matter between the respective electrodes. Air has a much higher impedance than, for instance, blood. With the application of tomography, the sequential signals can be built up in to a 2 dimensional 32 × 32 pixel picture in real time with a frame rate of between 20 and 50 Hz^23^. The information is visualised on a screen as a “heat map”, where areas of impedance change (equivalent to ventilation) are displayed.

The signal gives information on both real time, regional, global, and relative data. With the correct application, and understanding of the monitor, much clinical information can be gained, with potentially significant patient benefit.

The information can be categorised in to global chest expansion, regional ventilation, and ventilatory compliance (expansion over time), with both absolute and relative figures for all^23,24^. Interpreting this data requires an understanding of ventilatory physiology and experience, however is easily learnt by interested parties.

There are two main views: a live view which shows moving images of a cross section of the lung, with regional ventilation clearly seen, and an end expiratory lung impedance view, which gives breath by breath impedance of the whole lung.

Until now, this technology has mainly been clinically used, infrequently, in adults. We describe the clinical applications of the use of EIT in children, with the physiological background and interpretation of the images. We used a Draeger Pulmovista 500 EIT monitor, with a variety of belts which allow monitoring of children above 3.5 kg in weight.

The use of the monitor was explained to all parents and verbal consent obtained. The chair of the Nottingham 1 research ethics committee confirmed the United Kingdom Health Research Authority decision tool opinion, which states that this project is exempt from needing ethical approval.

### Clinical scenarios

EIT is used routinely in complex children on our PICU. There was no specific case selection; we present six cases where the use of EIT has been of direct clinical benefit to children being treated for a variety of conditions.

We present six differing clinical scenarios; all commonly found on Paediatric Intensive Care Units, and demonstrate how EIT can add important information which would not be otherwise available. We display the use of EIT in:

- Asthma
- Ventilation weaning and expansion recoil
- Sequential lobar collapse
- Targeted physiotherapy
- Pleural fluid assessment
- PEEP optimisation

### Asthma: evaluating dynamic hyperinflation and air trapping

#### EIT parameter: End-expiratory Lung Impedance

An 11 year old boy with status asthmaticus, ventilated on pressure control mode, (Peak Inspiratory Pressure (PIP) 25 cm H_2_O; Positive End Expiratory Pressure (PEEP) 4 cm H_2_O; Respiratory Rate (RR) of 30 and FiO_2_ 71%) with a clinical suspicion of air trapping and dynamic hyperinflation, was connected to the EIT. Ventilatory changes were attempted with the aim of optimising his gas exchange and reducing air trapping. It was unknown whether air trapping was occurring, relative to baseline.

**Figure 1:**
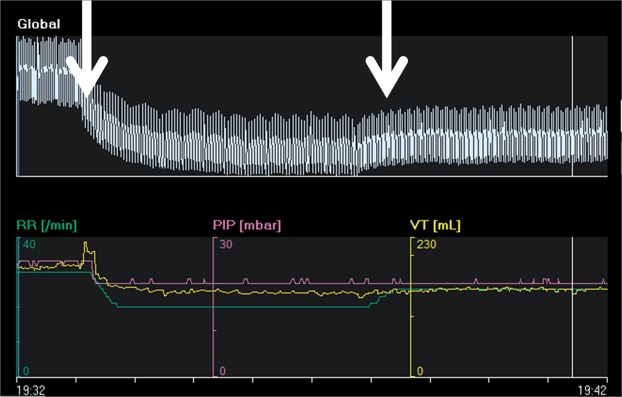
EIT global impedance in an asthmatic child.

The EIT trace shows initially a change from a PIP of 25 cm H_2_O to a PIP of 20, with constant PEEP, and a change of rate from 30 to 20 (Fig. 1, first arrow). The global impedance curve immediately begins to fall, reaching steady state around two minutes after. This signifies an overall decrease in the chest expansion (or end-expiratory lung volume [EELV]). This reduction in EELV is a strong indicator for a significant reduction in gas trapping, which should improve chest compliance.

At 6 minutes in to this recording (second arrow) the rate was increased to 25 due to a significant reduction in the minute volume. There was a small, but insignificant rise in the global expansion. The expansion remains lower than at the initial settings. Tidal volumes remained static, implying no change in the compliance (and no change in overdistension) after this rate increase. This allowed increased CO_2_ clearance with no corresponding hyperinflation at reduced ventilatory settings.

### Weaning of ventilation and extubation, with the use of expansion recoil

#### EIT parameter: Relative Global Chest Impedance/End-expiratory Lung Impedance

**Figure 2:**
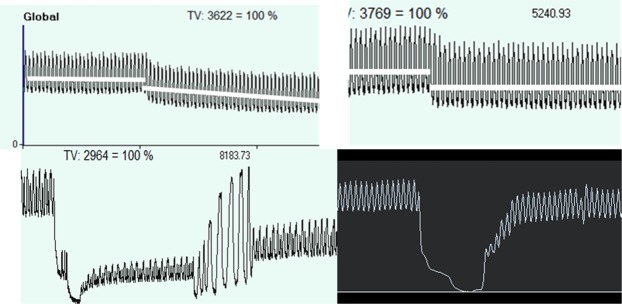
Relative global chest impedance on ventilatory weaning, and global expansion after suctioning. On the left after PEEP weaning there is sequential volume loss, and after suctioning there is an inability to regain previous expansion. On the right after PEEP weaning there is volume stability, and a rapid restitution of chest volume after suctioning.

A child with a diagnosis of pneumonitis whilst on volume ventilation was considered for weaning of ventilation as the ventilatory parameters had improved. However, when the PEEP was reduced by 2 cm H_2_O from 10 to 8, the global chest expansion displayed a sequential loss of volume over time. The PEEP was returned to the previous settings. On suctioning and returning to the ventilator, his oxygen saturations fell, requiring ventilation by anaesthetic bag. Analysis of the EIT trace showed that on returning to his ventilation, his global expansion (EELV) took a long time to return to baseline (Fig. 2).

After another period of ventilation, a similar reduction in PEEP showed a reduction in volume (as would be expected with reduced pressures), but this was stable over time^25^. He was suctioned for secretions and had not shown instability. Analysis of his EIT trace showed return to near baseline within a small number of breaths.

EIT was thus able to illustrate a lack of resilience to initial weaning, which later improved, providing added confidence to continue weaning towards extubation.

### Sequential lobar collapse: effect of postural changes

#### EIT parameter: live regional ventilation distribution/Tidal image

A 2 year old girl admitted with adenovirus and respiratory syncitial virus pneumonia with type 1 respiratory failure, ventilated on pressure control with PIP 30, PEEP 10, RR 25–30, and FiO2 0.7–0.8. She had frequent lobar collapse and she was monitored with EIT which showed right sided lung collapse. She was nursed with the chest right side up, and within one hour her ventilation was balanced and equal, with expansion returning to the collapsed lung. However, after another hour the left (dependant) side lost ventilation. For the next 48 hours, continuously monitored by EIT, she was repositioned as soon as one lung showed signs of loss of ventilation, usually every 3–4 hours. With this technique, the position of the patient was optimised based on lung expansion, and allowed the patient to wean ventilation and be extubated 3 days later.

**Figure 3:**
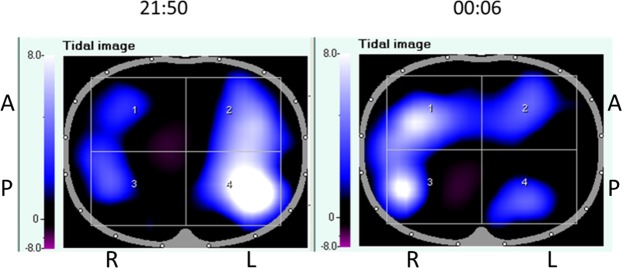
Live tidal image views showing sequential lobar collapse. Live tidal EIT images show the impedance change in each area, with brighter (whiter) pixels denoting a greater impedance change, equivalent to increased ventilation, in that area.

Figure 3 shows tidal images with a relative hypoventilation of the right lung, she was repositioned with the left side of the chest up and 3 hours later, the right lung expansion had improved, at the cost of the left lung ventilation. She was then turned again.

### Targeted Physiotherapy: Lobar or lung collapse: assessing progression and effect of physiotherapy

#### EIT parameter: Tidal image showing relative regional ventilation distribution

A 9 year old boy was admitted with a background of brainstem glioma when he was 2 years old, with significant sequelae including paraplegia and loss of brainstem function needing long term ventilation support 24 hours a day though a tracheostomy He was admitted with right sided total lung collapse needing PICU admission and escalation of his ventilation to a PICU ventilator: pressure control with PIP 22, PEEP 10, RR 20, and FiO2 80–100%.EIT monitoring was instigated. He received regular airway clearance using manual techniques and mechanical insufflation-exsufflation (MI:E).

**Figure 4:**
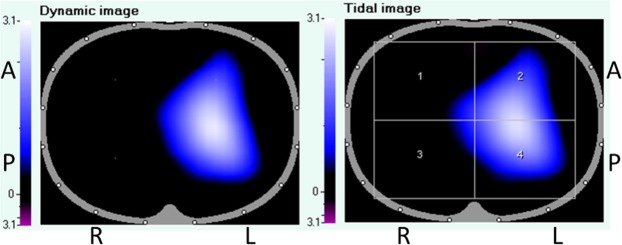
Live chest impedance views showing a complete lack of right sided expansion. Live tidal EIT images show the impedance change in each area, with brighter (whiter) pixels denoting a greater impedance change, equivalent to increased ventilation, in that area.

Observing EIT during physiotherapy treatment it was clear that the exsufflation component of MI:E, despite effectively clearing secretions, was causing temporary collapse of the right lung (Fig. 4). This was not resolved by the 2 re-inflation breaths available on the MI:E device. The physiotherapy team were able to adjust their treatment plan to include further re-recruitment following exsufflation. This prevented deterioration in objective parameters following airway clearance.

Following 6 days of treatment, his right lung was clinically fully reinflated, confirmed by EIT. He did not have any chest radiographs during this time.

### Pleural effusion: quantifying respiratory compromise

#### EIT parameter: relative regional ventilation distribution

**Figure 5:**
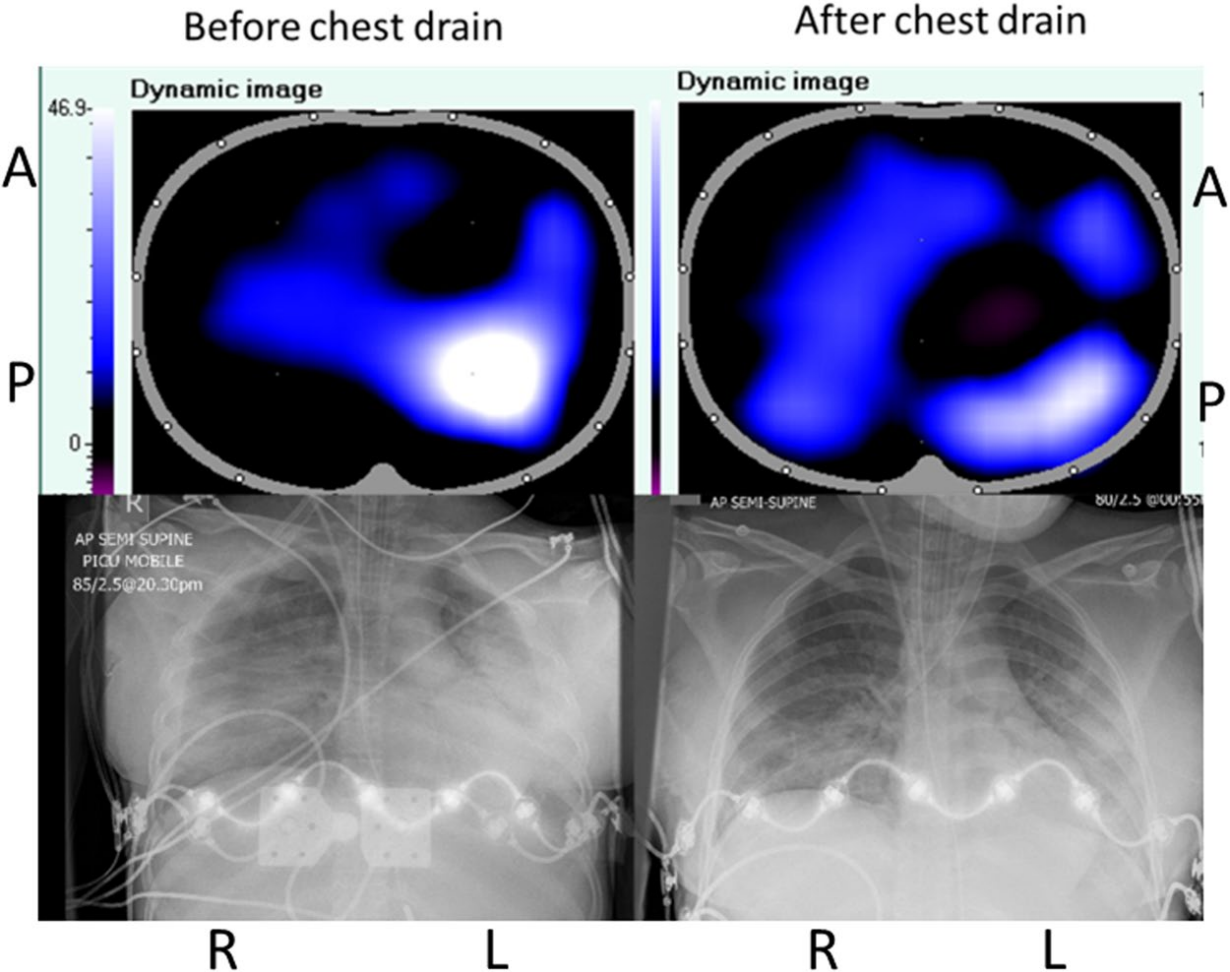
Pre and post chest drain live impedance images, with concurrent radiographs. Live tidal EIT images show the impedance change in each area, with brighter (whiter) pixels denoting a greater impedance change, equivalent to increased ventilation, in that area.

A 10 year old with a diagnosis of chronic myeloid leukemia present ed to the hospital with disseminated varicella zoster infection with multi organ failure needing respiratory, renal. and inotropic support. She had a pleural effusion of 1 cm on the chest x-ray, which was measured at 1.8 cm on chest ultrasonography. Her oxygen requirements were high, with an FiO_2_ of 0.8. 80%. A decision needed to be made whether insertion of a chest drain would be beneficial, with the heightened risk of an intrathoracic bleed due to the presence of disseminated intravascular coagulopathy. EIT showed a very significant loss of expansion in the affected side, compared to the non affected side. On balance, a chest drain was felt to be in her best interests, and it was inserted without complication. The ventilation improved. EIT pictures showed real time improvement of inflation, with aeration up to the chest wall (Fig. 5).

### PEEP optimisation

#### EIT parameter: PEEP analysis tool

A 15 week old baby was admitted with Respiratory Syncitial Virus Bronchiolitis. She was ventilated with a PEEP of 8 cm H_2_O in 40% oxygen, with tidal volumes of 6.5 ml/kgl. A PEEP trial, monitored by EIT, was performed with PEEP pressures between 14 cm H_2_O and 4 cm H_2_O in decrements of 2 cm H_2_O. As PEEP was increased, there were areas of overdistension (compliance loss at high pressure, in orange), however as PEEP was reduced, there were areas of collapse (compliance loss at low pressure, in white)^26^. Choosing the optimal PEEP level minimises overinflation and reduces collapse and makes physiological sense.

**Figure 6:**
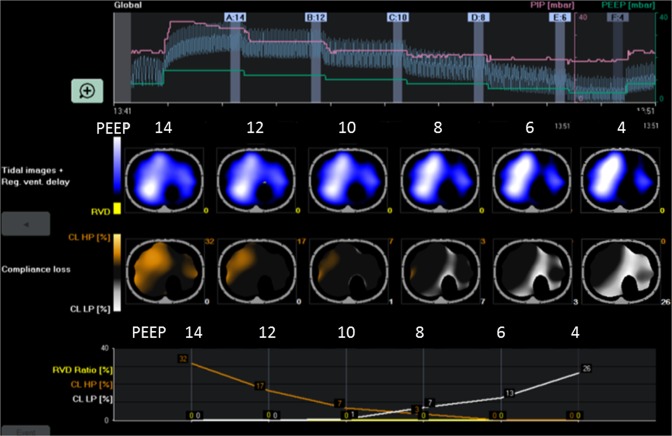
PEEP trial analysis showing overdistension and collapse at PEEP levels between 14 and 4 cm H_2_O. Global impedance is shown at the top at the six PEEP levels. Tidal images are shown in blue and white. Orange areas show areas of overdistension, and white areas of collapse. These percentages are shown on the final chart at the varying PEEP levels.

Analysis showed that the optimum PEEP (i.e. that which balances overdistension and collapse) was 9 cm H_2_O (Fig. 6). The patient’s PEEP was increased to 9 cm H_2_O.

## Discussion

We have presented six clinical scenarios where the use of EIT has materially benefitted our Paediatric Intensive Care patients. The information obtained by using EIT is difficult or impossible to be gathered by any other non-invasive technique. Conventional monitoring modalities give some feedback, but this is often delayed, inaccurate, or with a low level of accuracy^27^.

The interpretation of the images is critical to the use of EIT. Our six scenarios can be interpreted as follows:

1. Asthma: Gas trapping is a difficult problem in such patients and quantifying the extent of the problem is impossible. It only becomes apparent when ventilation becomes compromised, and then it is difficult to know whether this change in ventilation is due to clinical deterioration, or the respiratory dynamics. Use of EIT gives instant, breath by breath feedback on the global chest expansion, allowing pre-emptive management of incipient gas trapping problems.
2. Ventilation weaning and effects of disconnection. The patient will not display clinical signs soon after a ventilator change: it may take many hours for this to manifest. However, it can be anticipated that problems will arise if the patient is unable to maintain adequate lung expansion with the ventilatory settings which have been applied. If the global end-expiratory lung volume keeps dropping after the (expected) initial volume loss, then problems can be anticipated in the short to medium term. A patient who can hold a stable expansion after a ventilatory parameter change is likely to have a greater chance of medium term stability. Current monitoring techniques are poor at predicting rapidity of atelectasis and lung collapse after weaning. Knowledge of this may help to predict whether a patient is ready for extubation. The first trace shows a patient whose lungs cannot yet cope with a loss of PEEP, and is not ready for extubation. The second shows a patient who can rapidly restore lung volume and maintain adequate expansion making him suitable for extubation.
3. Sequential lobar collapse. Repositioning a patient with unilateral lobar collapse can cause respiratory instability, leading to a practice of minimal handling and reducing the frequency of unstable episodes. Clinical examination is inaccurate in a small chest with many added sounds. Chest radiography gives good knowledge of lobar collapse, but that may lead to worsening of gases and there is the added concern of radiation dosage. The use of EIT allows for monitoring of lung inflation in real time and ensure that significant areas of dependent collapse are avoided limiting respiratory instability due to repositioning. Direct visualisation identifies lung collapse rapidly making reinflation easier.
4. Targeted Physiotherapy. Constant EIT monitoring allowed us to monitor lung expansion without resorting to frequent chest radiographs. The physiotherapy team were able to quickly identify an adverse effect of treatment and adjust their plan accordingly. With standard monitoring it may not have been apparent why objective markers were deteriorating following airway clearance.
5. Pleural effusion. Chest X-Ray, and sonography, show a depth of fluid but do not give a sense of how much this fluid affects the lung inflation. Using live regional ventilation information allows precise visual quantification of this, meaning that clinical decisions can be based on more accurate information. In this case, the chest drain was high risk, but with the addition of EIT data this risk was felt to be worth taking. Cases of pneumothorax or foreign body would have a similar EIT picture, with the ability to quantify which areas of lung are non-functional and to what degree.
6. PEEP optimisation. Establishing the optimal PEEP in invasively ventilated patients is a challenge; the balance between collapse and over distension is difficult to assess and no method has been shown to prevent Ventilator Induced Lung Injury. EIT can be used to evaluate the regional compliance changes at different levels of PEEP, measuring areas of collapse and over distention during a PEEP trial. Both collapse and overdistension are detrimental to the patient. Knowledge of the effects of the PEEP to the patient allows the clinician to titrate it to produce minimal detrimental effects.

EIT is often used for continuous monitoring. Real time visualisation of chest expansion leads to knowledge of why an acute respiratory compromise has occurred. The treating team can be instantly aware of where the problem lies.

We also use EIT for progressing ventilation in the complex patient. No other non-invasive monitoring modality will give instant feedback over ventilatory changes. Such patient’s fragility means that delayed feedback from blood gas analysis or clinical change would be potentially harmful. The use of EIT allows the treating team to make changes they would not have dared to otherwise make due to their fragility. By making quicker progress than anticipated we are able to minimise lung injury and maximise chances of recovery.

A further clinical use on our unit is as a feedback spirometer. Conscious patients of a suitable developmental age can respond well to visualisation of how to ensure that breathing is effective.

There are multiple modalities of assessing a patient on mechanical ventilation (Table 1). There is, however, no perfect modality as they all have their disadvantages. Clinical examination is a single event, low resolution method of understanding lung movement. The benefits of CT scanning are offset by the radiation exposure as well as the logistical difficulties involved in taking the patient to the scanner. 28 X-Rays have lower logistical difficulties and lower radiation, but they do not allow continuous investigation, or measurements of flow or dynamic expansion^23^. Ventilator spirometry feedback allows no regional data, but does have the benefit of allowing continuous data29. Lung ultrasound gives important and useful information, but is a non-continuous, low resolution modality.

**Table 1:** Comparison of various methods of lung examination.

Comparison of different methods of chest examination						
Examination method	Regional Ventilation	Angle of examination	Flow	Measurement	Data	Chest expansion
Clinical examination	Yes (≈6 areas)	Mainly coronal	Yes (low reliability)	Single	Dynamic	No
Chest X-Ray	Yes (2D, moderate resolution)	Coronal	No	Single	Static	Yes
Chest CT scan	Yes (3D, high resolution)	Transverse	No	Single	Static	Yes
Chest Ultrasound	Yes (≈6 pixels)	Mainly coronal	Limited	Single	Dynamic	Limited
Ventilator spirometry	No	N/A	Yes, global	Continuous	Dynamic	No
Impedance Tomography	Yes (2D, 900 pixels)	Transverse	Yes, high resolution	Continuous	Dynamic	Yes

Compared to these monitoring modalities, EIT has significant benefits. It is a continuous, high resolution monitoring which allows assessment of dynamic, expansion, and flow data.

With any novel monitoring technology, there is a learning curve for clinicians. Although EIT needs good education for treating staff, this is a very visual monitor which can give information even if the technology behind it is not well understood. Our experience is that after some initial scepticism, the bedside team rapidly adopted it as the benefits were explained.

There is presently little clinical evidence of the efficacy of EIT in practice. Good ventilatory practice, involving the avoidance of hyperinflation and/or collapse, is associated with better outcomes. The information from EIT monitoring should allow better ventilatory practice, however the translation of this in to clinical outcomes is as yet unproven.

Suitably sized EIT belts are not commercially available for paediatric patients and the use of individual electrodes is time-consuming and risks skin irritation. This severely limits the use in this population. Its use in non-sedated patients is also limited due to patient anxiety and movement, causing potential image artefacts. Studies have also shown a difference in skin impedance in neonates as compared to adults 30. Reconstruction algorithms in current EIT modalities also do not account for differences in chest shape or size for neonates5. However, as this technology becomes more widely used, and the information gained from it better understood, there will be more commercial pressure to ensure that patients of all ages and sizes are catered for. As clinical experience and knowledge increases, this may become a routine part of clinical care in complex children.

With all forms of highly individualised medicine, there are concerns as to its effectiveness. Population evidence allows excellent assessment of a treatment’s long-term effectiveness, for a population mean. However, population evidence cannot tell the clinician whether the treatment will work for the individual patient, merely that if they treated 100 patients, it would be of overall benefit to give such a treatment.

This monitor offers evidence to the clinician that the physiology has changed for the individual patient. It cannot tell the clinician whether this has long term, or outcome benefits. An excellent understanding of physiology is necessary to ensure that physiological benefits then translate to outcome benefits.

## Conclusion

Electrical Impedance Tomography is a clinically useful tool on the Paediatric Intensive Care unit. It allows monitoring of a patient’s respiratory function in ways which are not possible through any other means. An understanding of respiratory physiology will allow use of this information to improve patient outcomes.
